# Use of continuous glucose monitoring system in patients with type 2 mellitus diabetic during hemodialysis treatment

**DOI:** 10.1186/s13098-021-00722-8

**Published:** 2021-10-09

**Authors:** Marcelo Rocha Nasser Hissa, Priscilla Nogueira Gomes Hissa, Sérgio Botelho Guimarães, Miguel Nasser Hissa

**Affiliations:** 1 Centro de Pesquisas em Diabetes e Doenças Endócrino-Metabólicas/Clinica Popular Endocrinologia, Rua Monsenhor Furtado, 1438/103 - Rodolfo Teófilo, Fortaleza, CE 60430-350 Brazil; 2 Universidade Federal do Ceará, Crateús, Ceará Brazil

**Keywords:** FreeStyle Libre, Glycemic monitoring, Diabetes Mellitus Type 2, Continuous glucose monitoring

## Abstract

**Background:**

Studies highlight the inaccuracy of glycated hemoglobin (HbA1c) for the assessment of glycemic control in dialysis diabetics and suggest the use of continuous glucose monitoring (CGM) as an alternative. Of the CGMs, FreeStyle Libre^®^ is the most used in worldwide, but there is still no consensus on its use in dialysis.

**Method:**

A 3-week prospective study was performed with 12 patients comparing capillary and interstitial glucose during dialysis.

**Results:**

Comparing capillary and interstitial measurements, similar values were observed in pre-dialysis in the 1st week (184.1 ± 69.5 mg/dl and 173.1 ± 78.9 mg/dl, respectively, p = 0.303), in patients with body mass index less than 24.9 kg/m^2^ (214.2 ± 72.2 mg/dl and 201.3 ± 77.0 mg/dl respectively, p = 0.466), in those dialysis fluid loss less than 2 l (185.5 ± 82.6 mg/dl and 183.1 ± 94.0 mg/dl respectively and p = 0.805) and in those with hemoglobin greater than 12 g/dl (152.0 ± 35, 5 mg/dl and 129.5 ± 47.4 mg/dl respectively, p = 0.016). In the correlation of the capillary measurement with the interstitial sensor, it was observed that the proportions in the Clarke Error Grid of zone A, zone B, zone C, zone D and zone E were 62.5%, 27.1%, 0.0%, 10.4% and 0.0% respectively and in the Parkes error grid in zone A, zone B, zone C, zone D and zone E were 80.6%, 9.7%, 9.7% 0.0% and 0.0%, respectively.

**Conclusion:**

The mean absolute relative difference in dialysis patients is higher than the general population without end-stage renal disease. However, clinical decision-making based on the values measured by the system can be made with a good margin based on the correlation between interstitial and capillary measurements.

## Introduction

The prevalence of chronic kidney disease (CKD) has steadily increased worldwide. Diabetes is now considered the main cause of end-stage renal disease (ESRD), accounting for up to 89.7% of dialysis disease cases [1]. Diabetic nephropathy is one of the costliest complications for the health system because patients with this condition often evolve to dialysis, an expensive treatment. American data showed that expenditures on ESRD exceeded US$ 120 billion in 2017. As an aggravating factor, cardiovascular mortality grows in proportion to the decrease in glomerular function and among diabetics who start dialysis treatment, less than 20% survive after 5 years [1].

Glycemic control of chronic kidney patients on dialysis presents additional difficulties because both uremia and dialysis can affect insulin secretion and tissue insulin sensitivity. In these patients, increased insulin resistance, increased hepatic gluconeogenesis, impaired intracellular glucose metabolism, decreased insulin clearance and decreased insulin secretion potentiated by metabolic acidosis are observed. Such factors contribute to wide fluctuations in blood glucose levels and exogenous insulin requirements, which become even more impaired by the alteration of the pharmacokinetics of exogenous insulin and hypoglycemic agents and predisposition to asymptomatic hypoglycemia [2].

Hemoglobin A1c (HbA1c) is the established gold standard indicator for assessing long-term glucose control in diabetes. Patients undergoing dialysis treatment have erythrocytes with a reduced shelf life and often use erythropoiesis-stimulating agents to treat nephrogenic anemia. These agents, when increasing erythropoiesis, increase the proportion of young non-glycated erythrocytes, underestimating the calculated mean glycemia [3]. It also contributes to this underestimating the reduction in lifespan of erythrocytes. The latest Kidney Disease Improving Global Outcomes guidelines highlight the inaccuracy of HbA1c, and suggest the use of continuous monitoring devices as an alternative [4].

Other markers of diabetes control such as fructosamine and glycated albumin can prevent problems related to the half-life of hemoglobin and provide alternatives to HbA1c [5]. The limitation of these markers is the impaired accuracy resulting from the influence of serum albumin, uric acid and bilirubin levels for their determination [2] and they still need more robust clinical evidence to elucidate their applicability in all the stages of CKD [6].

Continuous glucose monitoring (CGM) is a critical clinical tool for the treatment and management of diabetes. A small glucose sensor is inserted under the skin to measure glucose in the interstitial fluid. Devices that perform continuous transcutaneous interstitial glucose monitoring provide a means to facilitate diabetes control, resulting in better HbA1c levels, less glucose variability, less frequent hypoglycemic episodes, better quality of life, and more lifestyle flexibility. Clinical trials have demonstrated the benefit to control type 1 [7] and type 2 diabetes [8].

In patients undergoing dialysis treatment, the use of CGM emerges as a promising tool for the assessment of glycemic control, allowing the tracking of the physiological dynamics of glucose in greater detail, both in relation to meals and hemodialysis sessions [9]. Some recent interventional studies show that the CGM is a useful tool to guide the management of insulin therapy in this population. In one trial, CGM-adapted insulin therapy resulted in a reduction in HbA1c levels from 8.4% at baseline to 7.6% after 3 months of follow-up [6]. Relative Absolute Mean Difference (MARD) is used to assess the accuracy of CGM sensors. International guidelines recommend that a MARD with good accuracy should be less than 10% [10]. It is important to note, however, that CGM is mainly studied in the non-dialysis population and that there have only been studies in very small populations on dialysis.

FreeStyle Libre (Abbott Laboratories©) is an interstitial CGM system that uses a glucose sensor inserted into the skin over the triceps and a portable touchscreen reader that is used to scan the device. The sensor automatically measures glucose every minute and readings are stored at 15-min intervals.

Experience with CGM in CKD patients on dialysis is limited [3]. There is currently no consensus on the best metric to assess the accuracy of monitoring by Freestyle in dialysis.

## Subjects and methods

### Objective

To compare pre and post dialysis measurements by CGM and SMBG measured twice per dialysis three times per week over 2 weeks with a total of 144 measurements. Pre and post-dialysis measurements were analyzed in different clinical situations, evaluating the mean absolute relative difference in each dialysis session, and analyzing the correlation of the interstitial measure with the capillary.

### Participants

Thirteen patients were evaluated for initial screening. In the 1st week, all were able to maintain the research. One patient after sensor placement was discontinued for having been transplanted before the end of follow-up. Eligibility criteria included type 2 diabetes with diagnosis time greater than 4 months, in dialysis therapy for at least 30 days, aged 18 to 80 years, with a body mass index of 22 to 40 kg/m^2^ and ability to perform all the tasks of the study. The exclusion criteria were following: (1) pregnant or nursing women; (2) known allergy to sensor adhesive (3) extensive skin lesions or scars that make it difficult for the sensor to adhere (4) infection or edema at the sensor application sites. (5) use of medication that could impair the sensor’s glucose measurement; (6) type 1 diabetes, monogenic diabetes, diabetes resulting from pancreatitis, or secondary forms of diabetes (Cushing’s syndrome or acromegaly); (7) acute metabolic conditions such as ketoacidosis and lactic acidosis or hyperosmolar coma in the past 6 months; (8) acute or chronic liver disease, evidence of hepatitis, cirrhosis or portal hypertension, or history of imaging abnormalities that suggest liver disease (except hepatic steatosis), such as portal hypertension and cirrhosis.

### Study design

This is a 3-week prospective exploratory study conducted at the Davita Meireles Dialysis Clinic/Diabetes Research Center/Department of Surgery, Hospital Walter Cantídio, Federal University of Ceará, Brazil.

The study consisted of a period of 3 weeks, with 1 week of screening with capillary blood glucose and 2 weeks of intensive monitoring period with Interstitial CGM and digital capillary blood glucose. In the 1st week, participants had capillary blood glucose measured at the beginning and end of the 3 weekly dialysis sessions (Monday, Wednesday and Friday or Tuesday, Thursday and Saturday) with the Accu-Chek Guide glucometer and Accu-Chek Active blood glucose control strips. In week 2, the CGM sensor was placed in the upper-posterior part of the arm contralateral to the arteriovenous fistula 60 min before the beginning of the dialysis session. Patients were instructed to always bring the monitor to dialysis sessions. To measure capillary blood glucose levels as a reference, all participants used the Accu-Chek Guide. Data from the FreeStyle Libre reader was downloaded using the FreeStyle Libre software program, version 1.0 (Abbott Diabetes Care).

The study protocol was approved by the local Ethical Committee (Comitê de Ética em Pesquisa envolvendo seres humanos (COMEPE) from Centro Universitário Christus - Unichristus) and all eligible candidates had to provide signed informed consent before enrolling in the study.

### Measurement protocol

At the beginning of each dialysis session, capillary blood glucose was measured and compared with the interstitial measurement with a simultaneous interstitial sensor. Capillary and interstitial glucose measurements were compared before and after dialysis at each hemodialysis (HD) session individually. It was also analyzed in delimited situations such as age, time on dialysis treatment, body mass index (BMI), fluid loss from dialysis ultrafiltrate, hemoglobin and HbA1c.

### Statistical analysis

In order to characterize the population of each, descriptive analysis of frequency measurements was performed when the variable was qualitative; and mean and its variations when the variable was quantitative. Quantitative variables were tested for normality using the Shapiro–Wilk test. For the independent variables, in the presence of normality, the homogeneity of the variance of the groups was evaluated using the Levene’s test. Given the homogeneity, the difference was tested using an independent t-test. In case homogeneity was not proven by the Levene’s test, the differences between the independent variables were calculated using the Welch test. The distinction between quantitative variables without normality was verified by the Mann–Whitney test.

The Mean Absolute Relative Difference (MARD) was used to assess the accuracy of the Freestyle Libre interstitial sensor. Clarke Error Grid and Parkes Grid grids for type 2 diabetes were used, associating the paired results of the Freestyle meter with that of the Accu-Chek Guide glucometer.

## Results

The study enrolled 13 patients to initiate interstitial CGM monitoring simultaneously with an Accu-Chek Guide glucometer. One patient was withdrawn for having been transplanted before the end of follow-up. Of the 12 patients included in the study, 11 were also hypertensive. 10 patients used insulin treatment and only 2 oral medications (linagliptin), none of the patients had residual urination. The baseline characteristics are described in Table 1.

**Table 1:** Clinical and laboratory data

	Study population n = 12
Sex (M:F)	(8:4)
Age (years)	66.8 ± 8.0
Time in dialysis treatment (years)	4.7 ± 3.4
BMI with dry weight in V-3 (kg/m^2^)	25.5 ± 2.7
Loss volume per session (l)	2.0 ± 0.5
Hematocrit (%)	36.7 ± 7.3
Hemoglobin (g/dl)	11.9 ± 2.2
Initial fasting blood glucose (mg/dl)	209.4 ± 66.6
Initial glycated hemoglobin (%)	7.3 ± 1.1
Intradialytic blood glucose variation (mg/dl)	52.9 ± 56.9
Hypoglycemia episodes in the 1st week	0

Data presented as mean with standard deviation

**Fig. 1:**
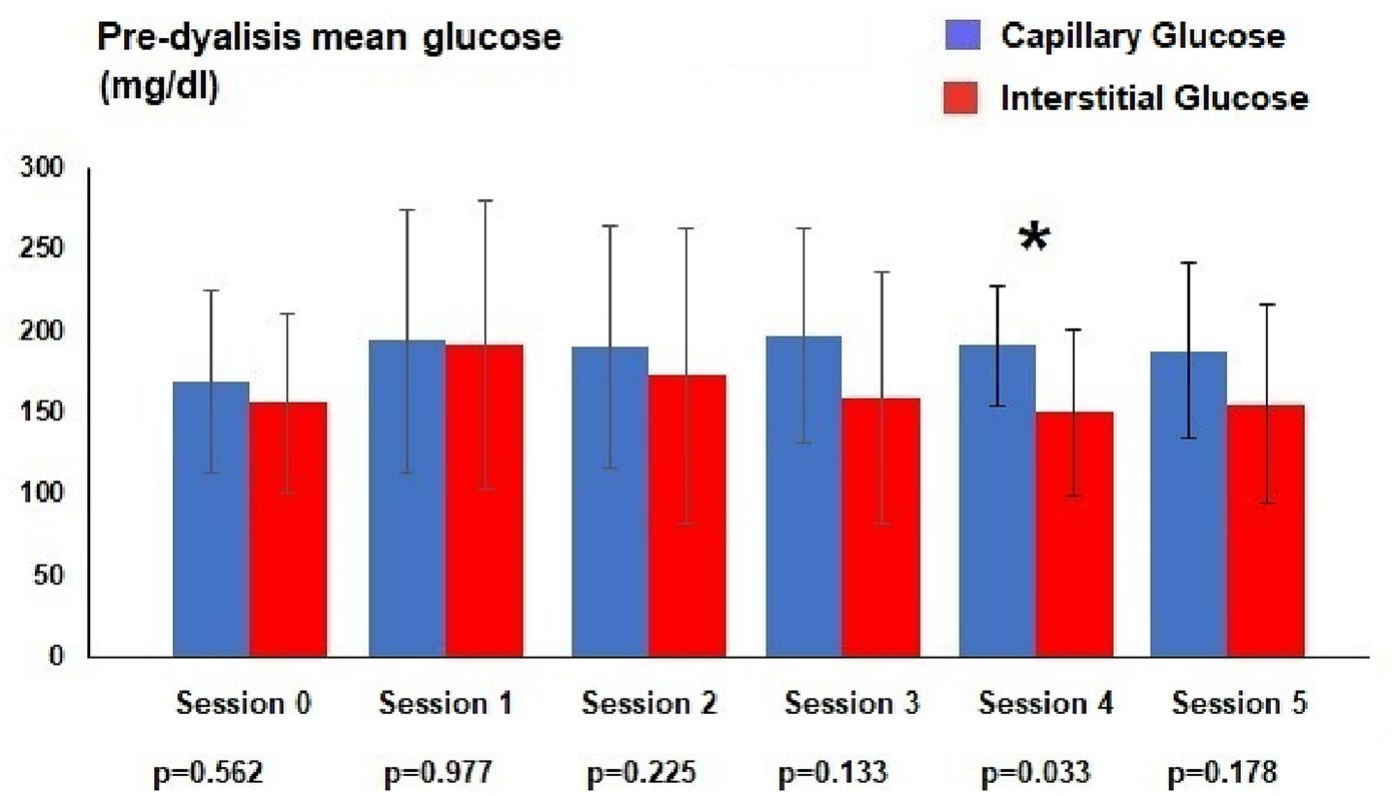
Average blood glucose in pre-dialysis (* statistical difference)

**Fig. 2:**
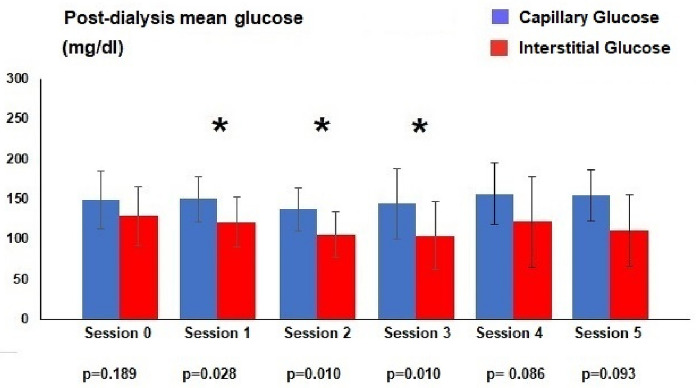
Average blood glucose in post-dialysis (* statistical difference)

Capillary blood glucose was similar to that of the interstitial sensor at HD session 0, 1, 2, 3 and 5. Only at session 4 there was a statistical difference between capillary and interstitial measurements (191.3 ± 36.3 mg/dl and 150.5 ± 50.6 mg/dl respectively, p = 0.033). Comparison of capillary and interstitial glucose measurements after dialysis showed more statistically significant differences. Capillary differences were observed at session 1 (149.6 ± 28.1 mg/dl and 121.1 ± 31.0 mg/dl respectively, p = 0.028), 2 (137.9 ± 26.8 mg/dl and 106.2 ± 28.0 mg/dl respectively, p = 0.010) and 3 (144.6 ± 44.3 mg/dl and 104.8 ± 42.1 mg/dl respectively, p = 0.010). There were also differences in the mean of all pre-dialysis capillary measurements with the sensor (188.3 ± 61.2 mg/dl and 163.9 ± 71.3 mg/dl respectively, p = 0.029) and with the post-dialysis measurements (148, 8 ± 34.0 mg/dl and 123.0 ± 46.0 mg/dl respectively, p = 0.000) (Figs. 1 and 2).

### Specific glycemic assessment

Some clinical and laboratory parameters were evaluated to determine which clinical situations the sensor measure matches with the capillary blood glucose measure. In pre-dialysis measurements, capillary blood glucose showed a discrepancy from the interstitial during the 2nd week of sensor use (192.6 ± 52.1 mg/dl and 154.7 ± 62.3 mg/dl respectively, p = 0.007), in patient with a BMI greater than 24.9 kg/m^2^ (162.4 ± 31.7 mg/dl and 126.6 ± 38.7 mg/dl respectively, p = 0.000), in patients who lose an average of more than 2 l in the dialysis ultrafiltrate (187.7 ± 48.1 mg/dl and 154.3 ± 55.4 mg/dl respectively, p = 0.002) and in patients with hemoglobin ≤ 12 (197.3 ± 52.0 mg/dl and 157 0.5 ± 56.1 mg/dl respectively, p = 0.006) (Table 2).

**Table 2:** Glucose measurements in specific pre- and post-dialysis clinical situations

	Pre-dialysis			Post-dialysis		
	Capillary glucose (mg/dl)	Interstitial glucose (mg/dl)	p	Capillary glucose (mg/dl)	Interstitial glucose (mg/dl)	p
1ª Week mean glucose (mg/dl) (n = 36)	184.1 ± 69.5	173.1 ± 78.9	0.303	145.4 ± 30.25	118.58 ± 32.7	**0.001**
2ª Week mean glucose (mg/dl) (n = 36)	192.6 ± 52.1	154.7 ± 62.3	**0.007**	152.3 ± 37.6	127.5 ± 56.4	**0.032**
Body mass index						
> 24.9 kg/m^2^ (n = 36)	162.4 ± 31.7	126.6 ± 38.7	**0.000**	152.9 ± 30.8	113.8 ± 38.9	**0.000**
≤ 24.9 kg/m^2^ (n = 36)	214.2 ± 72.2	201.3 ± 77.0	0.466	144.8 ± 37.0	132.3 ± 51.1	0.237
Water loss (ultrafiltrate)						
> 2 l (n = 48)	187.7 ± 48.1	154.3 ± 55.4	**0.002**	144.9 ± 33.5	112.6 ± 43.5	**0.000**
≤ 2 l (n = 24)	189.5 ± 82.6	183.1 ± 94.0	0.805	156.8 ± 34.3	143.8 ± 44.7	0.265
Age						
> 65 years (n = 42)	205.9 ± 68.7	177.1 ± 82.6	0.086	144.3 ± 30.2	122.2 ± 48.1	**0.014**
≤ 65 years (n = 30)	163.7 ± 37.7	145.5 ± 46.8	0.104	155.2 ± 38.4	124.2 ± 43.6	**0.005**
Time on dialysis treatment						
> 4 years (n = 36)	188.3 ± 56.6	164.9 ± 53.9	0.078	157.9 ± 39.6	131.7 ± 45.9	**0.012**
≤ 4 years (n = 36)	188.4 ± 66.2	162.9 ± 86.0	0.164	139.8 ± 24.7	114.3 ± 45.1	**0.004**
Hemoglobin						
> 12 (g/dl) (n = 42)	181.9 ± 66.9	168.6 ± 80.7	0.412	152.0 ± 35.5	129.5 ± 47.4	**0.016**
≤ 12 (g/dl) (n = 30)	197.3 ± 52.0	157.5 ± 56.1	**0.006**	144.5 ± 31.9	113.9 ± 43.2	**0.003**
Glycated hemoglobin						
> 7% (n = 42)	202.6 ± 72.0	167.6 ± 88.3	0.050	152.3 ± 35.9	119.2 ± 52.7	**0.001**
≤ 7% (n = 30)	168.3 ± 33.4	158.8 ± 37.0	0.303	144.1 ± 31.2	128.4 ± 34.7	0.072

Data presented as mean with standard deviation. p significant when < 0.05. The n in parentheses correlates with number of measurements

Regarding post-dialysis measurements, capillary blood glucose measurements were different from those measured by the interstitial sensor in the 1st week (145.4 ± 30.25 mg/dl and 118.58 ± 32.7 mg/dl respectively, p = 0.001) and in the second of treatment (152.3 ± 37.6 mg/dl and 127.5 ± 56.4 mg/dl respectively, p = 0.032), in a patient with a BMI greater than 24.9 kg/m^2^ (152.9 ± 30.8 mg/dl and 113.8 ± 38.9 mg/dl respectively, p = 0.000), in patients who lose more than 2 l in the dialysis ultrafiltrate on average (144.9 ± 33.5 mg/dl and 112 0.6 ± 43.5 mg/dl respectively, p = 0.000), in patients older than 65 years (144.3 ± 30.2 mg/dl and 122.2 ± 48.1 mg/dl respectively, p = 0.014) and under 65 (155.2 ± 38.4 mg/dl and 124.2 ± 43.6 mg/dl respectively, p = 0.005), in patients with more than 4 years of dialysis treatment (157.9 ± 39, 6 mg/dl and 131.7 ± 45.9 mg/dl respectively, p = 0.012) and there is less (139.8 ± 24.7 mg/dl and 114.3 ± 45.1 mg/dl respectively, p = 0.004), in a patient with hemoglobin greater than 12 g/dl (152.0 ± 35.5 mg/dl and 129.5 ± 47.4 mg/dl respectively, p = 0.016) and smaller (144.5 ± 31.9 mg/dl and 113.9 ± 43.2 mg/dl respectively, p = 0.003) and glycated hemoglobin greater than 7% (152.3 ± 35.9 mg/dl and 119.2 ± 52.7 mg/dl respectively, p = 0.001) (Table 3).

**Table 3:** Percentage of points in Clarke and Parkes Grid zones

Percentage of points in Clarke and Parkes Grid zones, in pre, post-dialysis and overall					
	Zone A	Zone B	Zone C	Zone D	Zone E
Pre-dialysis Clarke Grid (%)	72.2	22.2	0	5.6	0
Post-dialysis Clarke Grid (%)	52.8	31.9	0	15.3	0
Overall Clarkes Grid (%)	62.5	27.1	0	10.4	0
Pre-dialysis Parkes Grid (%)	87.5	4.2	8.3	0	0
Post-dialysis Parkes Grid (%)	73.6	15.3	11.1	0	0
Overall Parkes Grid (%)	80.6	9.7	9.7	0	0

Percentage of points in each specific area of the graph with pre, post-dialysis and total measurements

### Clinical verification

The mean relative absolute difference (MARD) was calculated using capillary blood glucose as a reference. The MARD of each hemodialysis session was compared with that of session 0 at the beginning and end of the dialysis session. Regarding pre-dialysis values, a progressive increase in MARD from the first session during each measurement was observed, reaching a statistically significant peak in session 3 (p = 0.0013) and followed by a subsequent decrease until session 4 and 5 (9.4 ± 6.3%, 11.2 ± 12.8%, 15.3 ± 16.5%, 23.6 ± 17.0%, 21.5 ± 19.0% and 21, 1 ± 21.8% respectively).

**Fig. 3:**
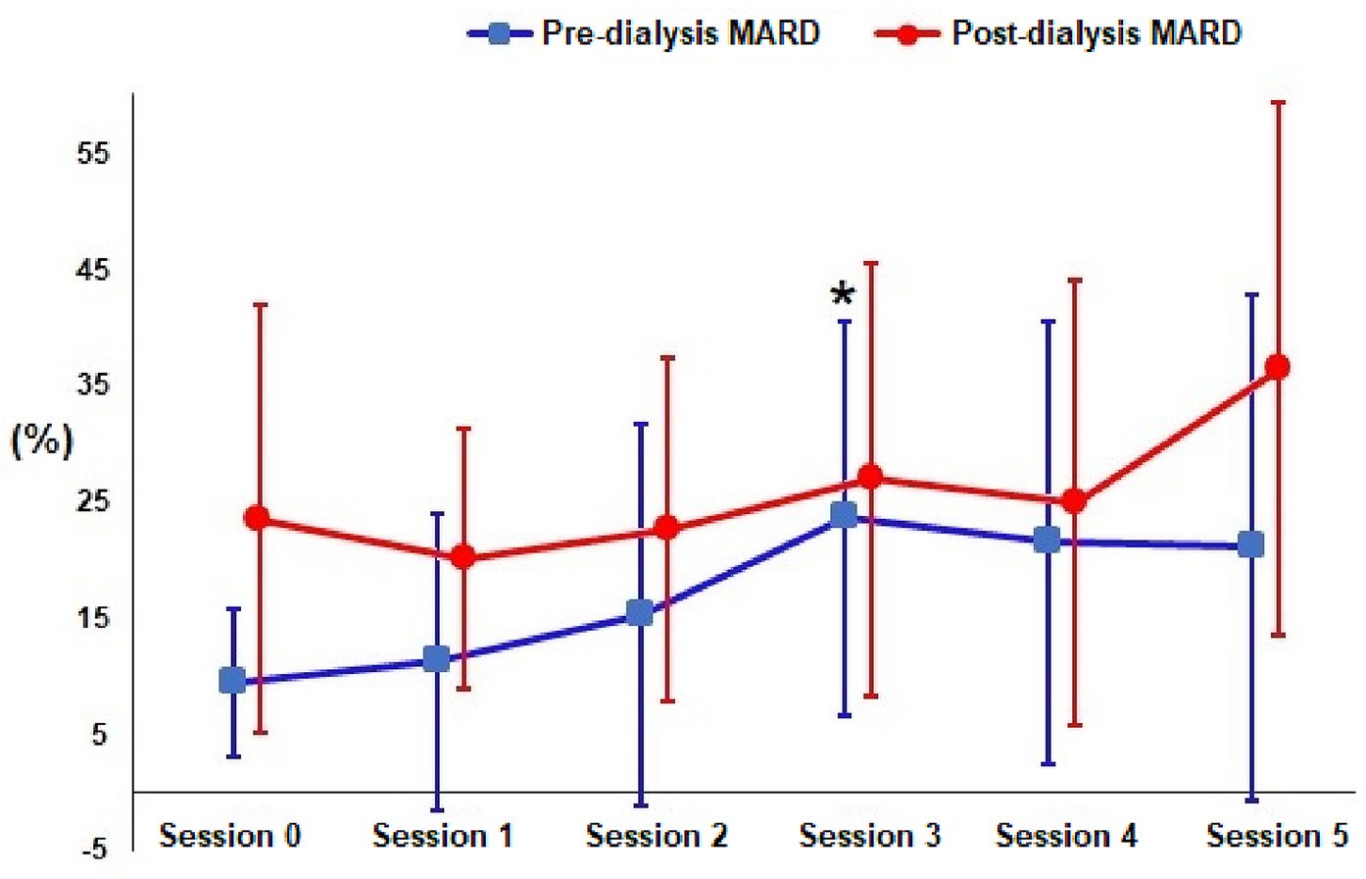
MARD of each dialysis session (*statistical difference in relation to the reference measure V0)

The MARDs calculated in the post-dialysis showed a higher value in the first session with a drop in the subsequent session followed by a progressive increase, however without statistically significant differences throughout each session (23.5 ± 18.4%, 20.1 ± 11, 3%, 22.6 ± 14.8%, 26.9 ± 18.6%, 24.9 ± 19.2% and 36.4 ± 22.9% respectively). In the mean MARD per session without distinction between pre- and post-dialysis, the last session was the only one that showed significant differences from the first (p = 0.037). The MARD with all values gathered was 21.4% (± 17.8) (Fig. 3).

**Fig. 4:**
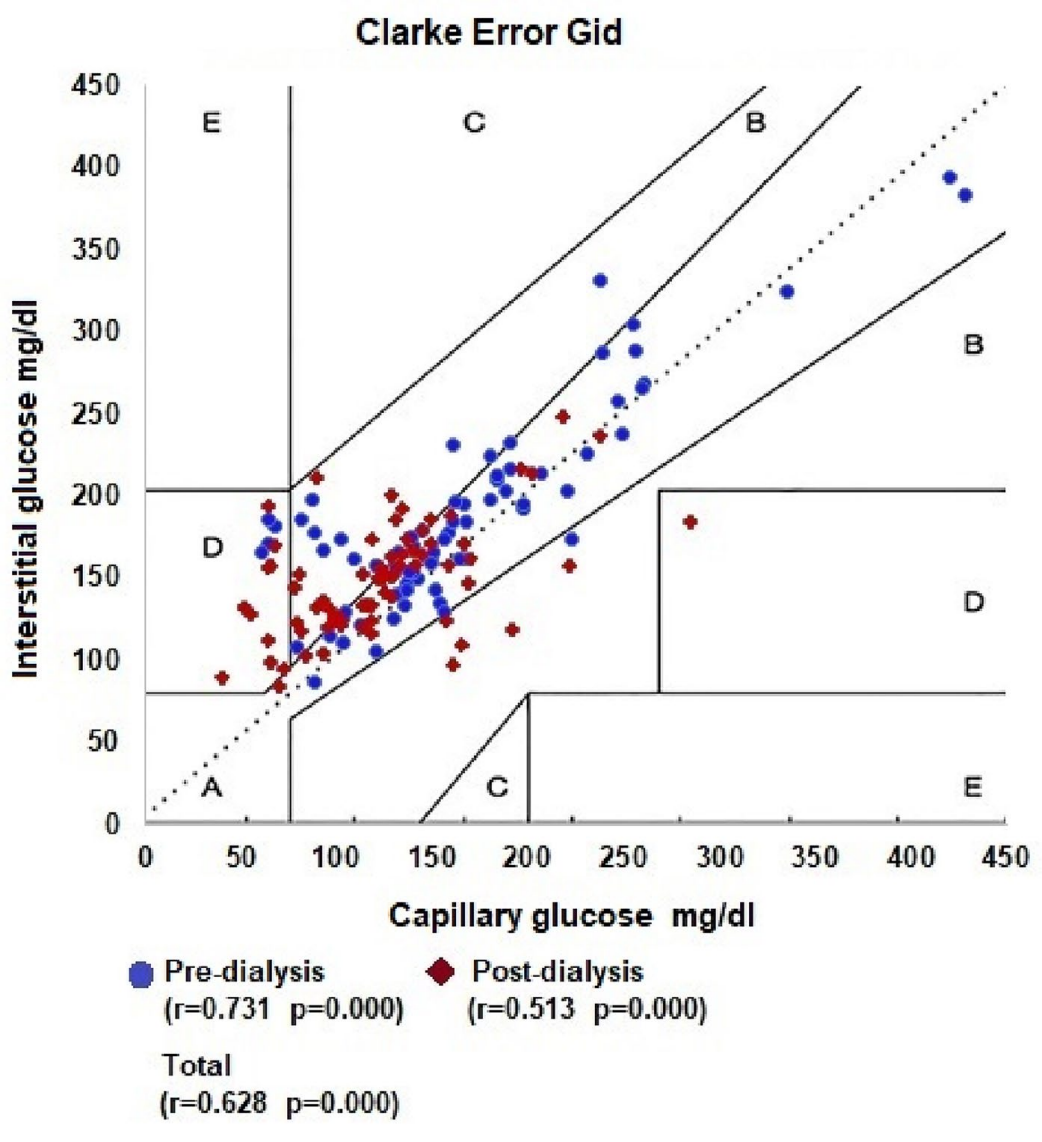
Clarke Error Grid overall values per dialysis session

Regarding the Clarke Error Grids, it was observed in the correlation with pre-dialysis measurements, the proportions of zone A, zone B, zone C, zone D and zone E were 72.2%, 22.2%, 0.0%, 5 0.6% and 0.0%, respectively. In the correlation with post-dialysis measurements, the proportions of zone A, zone B, zone C, zone D and zone E were 52.8%, 31.9%, 0.0%, 15.3% and 0. 0%, respectively. Considering all measurements, the proportions of zone A, zone B, zone C, zone D and zone E were 62.5%, 27.1%, 0.0%, 10.4% and 0.0%, respectively (Table 3 and Fig. 4).

**Fig. 5:**
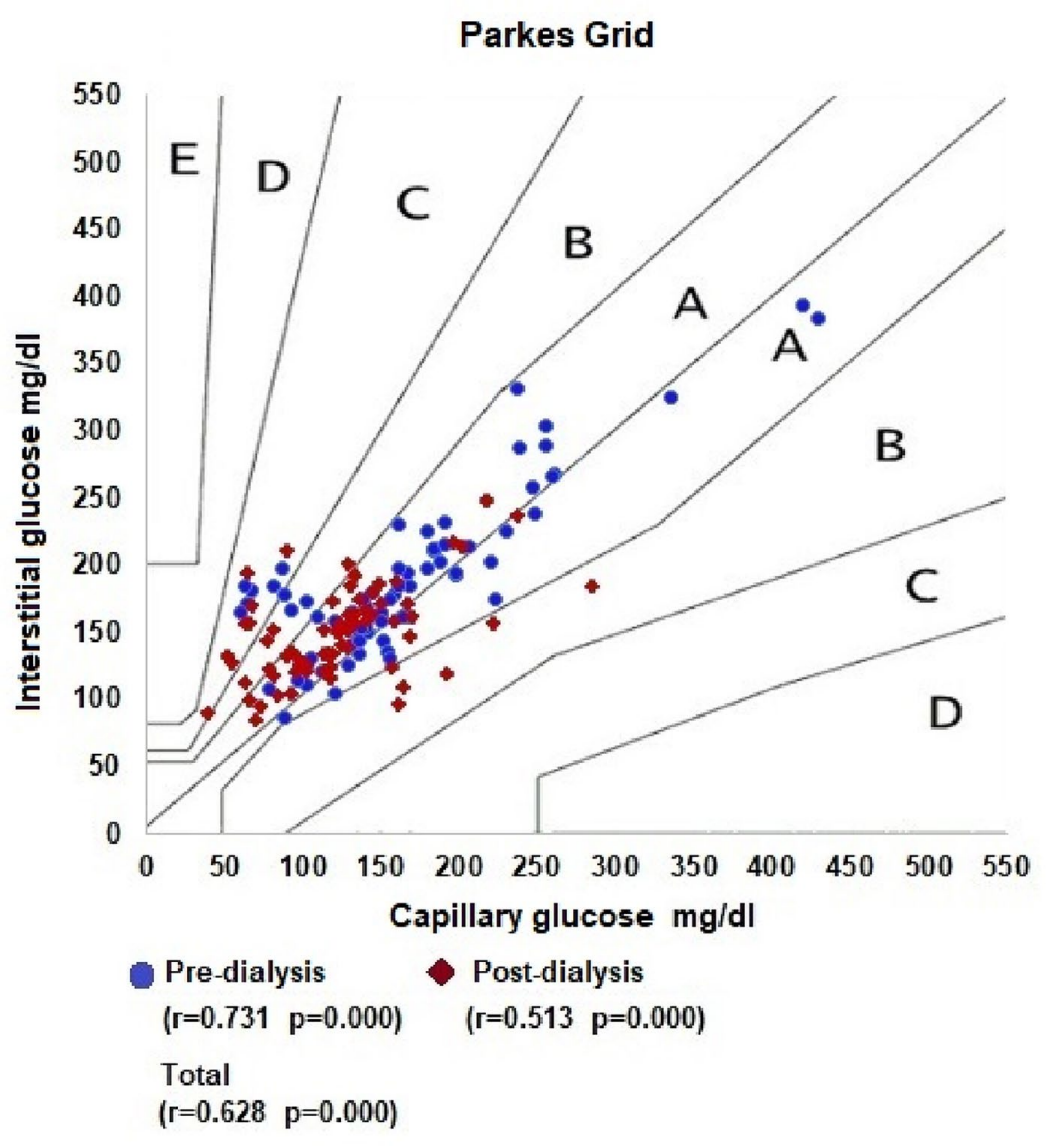
Parkes grid overall values per dialysis session

In the Parkes grid measured in pre-dialysis, the proportions of zone A, zone B, zone C, zone D and zone E were 87.5%, 4.2%, 8.3% 0.0% and 0. 0%, respectively and in post-dialysis, the proportions of zone A, zone B, zone C, zone D and zone E were 73.6%, 15.3%, 11.1% 0.0% and 0. 0%, respectively. In the association of overall measures, the proportions of zone A, zone B, zone C, zone D and zone E were 80.6%, 9.7%, 9.7% 0.0% and 0.0%, respectively (Fig. 5).

## Discussion

CGM emerges as a promising tool for patients who want to avoid large glycemic variability in the dialysis session [11]. Some studies have already pointed to an improvement in the management of type 2 diabetes who are undergoing hemodialysis with CGM [12], but it is still not clear whether the use of CGM can improve blood glucose and control or reduce the risk of hypoglycemia [3].

Our results confirm similar findings from another study regarding the bias of obtaining accurate interstitial glucose results in patients undergoing dialysis treatment [13]. At the beginning of each session, the difference between the capillary blood glucose values and the glucose obtained by the interstitial sensor was not significant, with the exception of visit 4. These results contrast with those obtained at the end of dialysis when in only half of the visits the sensor showed equivalent results the capillary measurement. It is noteworthy that inequality did not show a temporal pattern, that is; the differences were independent of the progression of visits. A possible explanation for the dual pattern of pre and post-dialysis measurements may be the changes in of extracellular fluid volume due the dialysis impairing the assessment of the interstice, as assumed by other researchers [14].

Some specific clinical situations result in better sensor accuracy. At the beginning of each session, the results are more similar between the capillary and the sensor when obtained in the 1st week of use, as well when obtained in patients with a BMI < 24.9 kg/m^2^, in patients who lose less than 2 l in the ultrafiltrate, in the absence of anemia (Hb ≤ 12 g/dl). In post-dialysis measurements, only those in patients with a BMI < 24.9 kg/m^2^, in those with fluid loss less than 2 l and in those without anemia, similarity with the capillary measurement is maintained. We emphasize that this sub-analysis provides more inaccuracy as the number of available measurements decreases.

We also carried out the clinical verification of the interstitial sensor in order to provide data that authorize clinical decision based on the values shown on the sensor monitor. The most commonly used metric for this purpose is the mean absolute relative difference (MARD). The lower the MARD the better the accuracy of the system. There is still little evidence of the accuracy of interstitial glucose sensors in the context of hemodialysis. Our results demonstrate that the MARD at the beginning of each dialysis session has the lowest values in the first session and rises to the apex in fourth session with a modest subsequent descent. In post-dialysis, all MARDs were higher than in pre-dialysis, with higher values in last session of use.

Our results showed MARD values between 16.5 and 19.0% in the 1st week, and in the 2nd week, values ranged between 25.3 and 28.8%. These values corroborate another study carried out in diabetics undergoing dialysis therapy whose MARD in the first week ranged from 13.8 to 21.0%, and in the second it ranged between 24.5 and 36.1% [13]. The progressive increase in MARD over days of use can be explained in part by the body’s natural inflammatory response to sensor insertion, which has been shown to affect the concentration of glucose in the interstitial fluid [15]. We cannot also rule out some factors that can influence the accuracy of the MARD and should be properly investigated in future studies, such as: weight changes between dialyses, blood flow, dialysis fluid flow, membrane area, type of dialysate (glucose concentration).

The total MARD in our study without distinction of pre- and post-dialysis measures was 21.4%, similar to the finding of a study in patients also on dialysis that demonstrated an overall MARD of 19.5% [16]. The use of Freestyle in patients without dialysis chronic kidney disease demonstrated a stable MARD pattern over the days, which varies depending on the study evaluated; from 10% [17], from 11.4% [18] to 16.8% to 17.8% [19].

Regarding the Clarke and Parkes error grid, our results differ from the results observed in the literature involving patients with chronic kidney disease on dialysis. Our sample of 144 measurements resulted in 90.3% of patients in Zone AB of Parkes and 89.6% of Clarke. Another study with a smaller sample (104 measurements) showed a percentage of 100% in the same area of Parkes [16]. A larger study (n = 2885 measurements) resulted in 99.7% in Clarke’s AB zone and 99% in Parkes [13]. However, our findings in zone A were 80.6% in the Parkes grid against 39.8 in the study by Toyoda et al. The lower frequency in the AB zone of our study can be partially attributed to the fact that, as we only used measurements taken during dialysis, we eliminated the bias of interdialytic blood glucose. As during dialysis there is great manipulation of fluid volume, there is greater probability of finding discrepancies between capillary and interstitial measurements.

Our data show less sensitivity to identify patients in zone AB and more specificity for patients in zone A alone (Table 4). We can assume that these findings can be explained by the influence of uremia, chronic inflammation and acidosis. We do not rule out or other unanticipated factors, however more study is needed to scrutinize such possibilities. It is important to point out that despite the frequencies mentioned above, the correlation coefficient between the measurements of capillary blood glucose and the CGM, despite being positive, was only moderate.

**Table 4:** Comparison of the percentage of points in error grid with other studies in the literature

Clark and Parkes error grid percentage	Zona AB (%)	Zona A (%)
Clarke Error Grid		
Our results (n = 144)	90.3	62.5
Dialysis patients (n = 2885) [13]	99.7	41.8
Non-dialysis patients (n = 6696) [17]	99.5	88.8
Non-dialysis patients (n > 25.000) [18]	99	85.5
Parkes Grid		
Our results (n = 144)	89.6	80.6
Dialysis patients (n = 2885) [13]	99	39.8
Dialysis patients (n = 104) [16]	100	93.3
Non-dialysis patients (n = 6696) [17]	99.9	87
Non-dialysis patients (n > 25.000) [18]	99.7	86.7

Among the limitations of our study, we can mention the small sample, the collection of data from only a single dialysis clinic, the non-comparison of glycemic data with other control markers in dialysis patients such as the dosage of frutosamina and glycosylated albumin, the number limited number of sensors lots used in the study. Additional research should be undertaken to assess the clinical value of the system, in terms of its long-term use and improved health outcomes and reduced cardiovascular risk.

## Conclusion

The use of the interstitial glucose measurement system with an interstitial sensor presents some differences when compared to capillary measurements. Greater accuracy of the system can be obtained when used in at the beginning of the dialysis session, in the first week of use, in normal weight patients, in patients with dialysis fluid loss less than 2 l and in patients without anemia. Clinical decision based on the values measured by the system can be made safely as a result of the correlation between interstitial and capillary glucose measurement.

## Data Availability

The data that support the findings of this study are available from the corresponding author upon reasonable request.

## Abbreviations

HbA1c: Glycated hemoglobin
CGM: Continuous glucose monitoring
CKD: Chronic kidney disease
MARD: Mean absolute relative difference
BMI: Body mass index
